# Does repeatedly reporting positive or negative emotions in daily life have an impact on the level of emotional experiences and depressive symptoms over time?

**DOI:** 10.1371/journal.pone.0219121

**Published:** 2019-06-27

**Authors:** Hendrik-Jan De Vuyst, Egon Dejonckheere, Katleen Van der Gucht, Peter Kuppens

**Affiliations:** Department of Psychology and Educational Sciences, KU Leuven, Leuven, Belgium; Unviersity of Sheffield, UNITED KINGDOM

## Abstract

The extent to which people are aware of their emotional experiences, label them and communicate them to the outside world are considered to impact emotional experience itself and potentially people’s depressive symptom levels. All of these aspects are important elements of one of the most common methods to study and measure emotions in the context of daily life, the so-called experience sampling method (ESM). A straightforward question that arises when using this method is whether participating in ESM may bring about changes in the momentary emotional self-reports of the people engaging in it, thereby effectively influencing that what it intends to measure; emotional experience over time, and whether this would relate to average levels of depressive symptoms. To examine these questions, we conducted a 7-day ESM study in which 90 participants were randomly assigned to repeatedly report either positive emotions only, negative emotions only or non-emotional internal states only, course using smartphones. Participants also completed pre-, post- and follow-up measurements of levels of depressive symptoms. Results showed no significant impact on self-reported momentary emotions, respective to their condition, over time nor on average levels of depressive symptoms across groups. These findings suggest that the repeated assessment of emotions in daily life, over the course of seven days, does not impact their emotional experience over time nor levels of depressive symptoms.

## Introduction

The emotions people experience play a crucial role in determining their well-being and risk for psychopathology [1, 2]. However, not only which emotions, positive or negative, people experience, but also how we relate to these emotional experiences is considered to play an important role for psychological well-being [3]. How we relate to these emotional experiences greatly depends on the extent to which we are aware of them, label them and communicate them to the outside world.

### Relating to emotions through awareness, labeling and external communication

Research suggests that the act of generating awareness of one’s emotional world is a necessity for adaptive emotion regulation [4] and an important contributor to our general well-being [5]. Awareness of emotional patterns, for instance, helps us to recognize their recurrent situational nature, to which we can then modulate our reactions in an adaptive way [5–6].

Emotional awareness is also a prerequisite for verbalizing our emotional states, otherwise known as the act of affect labeling [7]. This act of incidental emotion regulation has repeatedly been linked with the downregulation of that particular state, hence reducing one’s emotional distress [7–10].

Further, labeling an internal state allows for the effective communication of it to others. This act of emotional disclosure has been strongly associated with beneficial changes in one’s psychological well-being through decreases in anxiety [11–12], anger [11], depression [12–14] and several physical symptoms [13–14].

As it turns out, these three aspects (awareness, labeling, and communication) of how we relate to our emotional experiences are major components of one of the most commonly used methods to study and measure emotions in daily life, namely the experience sampling method (ESM).

### The experience sampling method

ESM is the golden standard when it comes to studying the psychology of everyday life [15]. It involves asking participants to repeatedly provide momentary self-reports regarding their thoughts, feelings, behaviors and/or context information, typically using electronic devices whilst in their natural environments.

A great deal of ESM research focuses on the realm of emotional experiences and thereby utilizes questionnaires focused on participants’ emotional world [16]. Such questionnaires often involve questions about how participants feel right now (e.g. “To what extent do you feel [happy] right now?”, using a rating scale ranging from “*Not at all*” to “*Very [happy]*”), or over a recent period of time (e.g. “How [happy] did you feel since the last beep?”, using a rating scale ranging from “*Not at all*” to “*All the time*”). In other words, this method requires individuals to engage in the repeated momentary introspection by becoming aware, verbalizing and externally reporting on their emotional experiences.

This raises the important question of whether the processes invoked by such introspective activities associated with ESM studies can in some way affect the momentary emotional self-reports of the individuals engaging in them. Put differently, could merely partaking in ESM research, and thereby adhering to its emotion-focused protocol, alter the self-reported momentary emotions of the participating individuals over time, in ways similar to those that the processes of becoming aware, verbalizing and externally communicating on emotions are known to do?

#### Previous research on method reactivity to ESM

Previous research on reactivity associated with participating in ESM studies has been conducted and indications have been found that it may indeed exist, revealing both direct effects on the emotional experiences involved, as well as indirect effects on more stable components of psychological well-being such as depressive symptom levels.

In this regard, Conner & Reid [17] detected direct changes in levels of happiness during a 14-day happiness-reporting ESM procedure. Moreover, this effect was moderated by one’s levels of trait negative affect: individuals scoring low on this trait would report higher levels of happiness at the end of the ESM period compared to baseline and vice versa [17]. In two other studies, indirect changes in depressive symptoms were observed in participants enrolled in an ESM protocol. Broderick & Vikingstad [18]. observed a decrease in depressive symptoms in rheumatology patients participating in a 30-day ESM protocol questioning the severity of pain and fatigue. Similarly, Kramer et al. [19]. found a significant (but temporary) decrease in depressive symptoms after measuring current positive affect in depressed out-patients during a 6-week ESM protocol.

In contrast, in other studies no evidence for ESM reactivity was found. For example, Vachon et al. [20]. observed no changes in depressive symptoms when asking patients suffering from Major Depressive Disorder to report on their symptoms (including the presence of positive and negative emotions) for a five-month period.

Clearly, the above findings do not allow us to draw unequivocal conclusions regarding the existence of possible reactivity to ESM in terms of emotionality or symptoms of depression. In addition, all abovementioned studies simply looked at changes in various aspects of one’s psychological well-being as a function of merely taking part in ESM research but none of these studies were explicitly designed to address the question of whether reporting on emotions per se may affect this. Critically, in these studies, a control condition in which participants repeatedly reported on other, related but non-emotional, aspects of their experience was absent. Only the inclusion of such a relevant control condition allows us to make inferences about whether reporting on one’s emotions on a regular basis, as is done in ESM research, affects participating individuals’ feelings and well-being. It could for instance be that merely being involved in a study that takes a reasonably long period of time and in which you are asked to do things on a regular basis may already have certain emotion-related implications.

### The current study

To address the question concerning ESM reactivity on direct emotional experiences and depressive symptom levels, we set up a between-subject experiment involving ESM. In this experiment, participants took part in an ESM protocol in which they reported on either emotional or non-emotional internal experiences (inasmuch this is possible, see below) for 7 consecutive days, with the latter group serving as a control condition. Further, being the first study to do this, in the experimental conditions we made a distinction between participants reporting solely on positive emotional states and participants reporting solely on negative emotional states, providing us the possibility to further observe potential indicators of reactivity in light of these contrasting instructions. Thus, in short, the study involved three different between-subject conditions: A first in which participants reported on positive emotional experiences only (positive condition), a second in which participants reported on negative emotional experiences only (negative condition), and a third in which participants reported on non-emotional internal experiences only (control condition). In order to study changes in levels of depressive symptoms, we assessed participants’ depressive symptom levels before, after, and at one-month follow-up of the experiment.

As indicators of reactivity to ESM, we examined (1) changes in the trajectories of these condition-specific self-reported emotional experiences throughout the ESM week, as well as (2) changes in participants’ depressive symptom levels throughout the study. We expected (1) that partaking in the ESM procedure would entail significant beneficial changes in the trajectories of self-reported emotional experiences in both experimental conditions. More specifically, we predicted an increase in positive affect in the positive condition and a decrease in negative affect in the negative condition. These expectations were based on the reviewed literature indicating that becoming aware, labeling, and externally reporting on your feelings might indeed alter our psychological well-being in mostly beneficial ways. Contrarily, no significant changes were expected in the control condition. Furthermore, following the same line of reasoning as above, we expected to see (2) a decrease in levels of depressive symptoms throughout the study in both experimental conditions. Again, no significant change was expected in the control condition.

Addressing this question is important from the perspective of both someone partaking in ESM research as for the researchers themselves. If taking part in such research may have consequences for psychological well-being, possibly interventions could be designed on the basis of it. Also, for the researcher it is crucial to know whether using this method merely measures emotions of participants or whether in some way or another it may also influence them, which would have important implications for what can and cannot be concluded based research using the method (i.e. the measurement changing that what it intends to measure).

## Method

### Participants

The sample comprised 90 university students (77% female), between 18 and 24 years old (*M*_age_ = 18.9, *SD*_age_ = 1.4) who were randomly assigned to one of three conditions with equal gender distribution (for every condition: *n* = 30; 77% female). Seventy-three of those participants were bachelor students in psychology who participated in exchange for course credits. For these participants, a reduction in ESM compliance (i.e. < 75% of the measurement occasions completed) would be met with a reduction in these credits, providing them with an incentive to complete as many momentary questionnaires as possible. The remaining 17 participants, who learned about the study through local advertisement such as flyers and social media, took part in the study on a voluntary basis and thus received no remuneration. All participants provided informed consent prior to initiating the study. No exclusion criteria were used. This study protocol was approved by the Social and Societal Ethics Committee of KU Leuven, Belgium (G-2017 01 738).

### Measures

All participants enrolled in an ESM protocol and completed assessments of their depressive symptom levels at pre- (T1), post- (T2) and one-month follow-up (T3). Participants also completed a number of other questionnaires (measuring alexithymia and mindfulness) that will not be reported here (but do not yield contradicting results in any way).

#### ESM protocol

Participants were enrolled in a 7-day ESM procedure in which they were semi-randomly prompted ten times a day (the time between 10 AM and 10 PM was divided into ten equal intervals and one beep was randomly programmed in each interval) to complete a short questionnaire about their momentary internal state via a research-dedicated smartphone. We instructed participants to take the smartphone everywhere they went and they had to respond to each beep within 90 seconds or the survey would be missed.

Depending on the condition that participants were randomly assigned to, they were asked to report on either ten positive emotional states (positive condition), ten negative emotional states (negative condition) or ten also internal, but inasmuch as possible, non-emotional states (control condition). The items in each condition were selected on the basis of a pilot study in which respondents (*N* = 85) were asked to indicate the subjective valence of a total number of 37 words. These words covered both high and low arousal (positive and negative) emotional states, as well as internal non-emotional states. These were rated on a scale ranging from zero (very negative), to five (neutral), to ten (very positive). To create the items for the three conditions from these data, 10 items were selected that were rated (1) either most positive (P), negative (N) and neutral (C) in affect (*M*_P_ = 8.39; *M*_N_ = 2.14; *M*_C_ = 4.35) and (2) showed the smallest degree of between-rater variance based on these ratings (*SD*_P_ = 1.49; *SD*_N_ = 1.48; *SD*_C_ = 1.26). The final items for each condition can be found in Table 1.

**Table 1:** Specific items for each condition.

Positive condition	Negative condition	Control condition
Relaxed	Sad	Tingling
Happy	Angry	Awake
At ease	Anxious	Hungry
Satisfied	Bad	A dry throat
Good	Stressed	Serious
Hopeful	Depressed	Itchy
Peaceful	Dissatisfied	The urge to urinate
Cheerful	Hopeless	Sleepy
Proud	Unhappy	Thirsty
Joyous	Irritated	Cold

At each beep, items were presented in a random order and all items were answered on a visual slider scale ranging from 0 (*not at all*) to 100 (*very much*). Reliability estimates of these items were calculated according to Nezlek [21] and showed substantial internal consistencies for both the positive (α_person level_ = .87; α_measurement occasion_ = .98) and the negative (α_person level_ = .74; α_measurement occasion_ = .99) condition. Internal consistencies in the control condition on the other hand were, unsurprisingly, non-existent to substantial (α_person level_ = .001; α_measurement occasion_ = .94).

#### Depressive symptoms

We assessed participants’ depressive symptoms with a Dutch version of the 9-item Patient Health Questionnaire [22]. With this survey, participants rated the frequency of nine common depressive symptoms over the past two weeks (e.g. ‘little interest or pleasure in doing things”). Items were rated on a 4-point Likert scale ranging from 0 (*Not at all*) to 3 (*Nearly every day*). The PHQ-9 showed good internal consistency at all three time-points (α_T1_ = .79, α_T2_ = .79, α_T3_ = .81).

### Procedure

In an initial lab session (T1) we invited participants to the lab in small groups of approximately ten people and collectively explained the purpose and timeline of the study. Participants were told that the study was designed to measure, and thereby gain further insight in, individual emotional trajectories throughout the day. In this way no attention was brought to the process of reactivity, minimizing potential demand effects. After providing written informed consent, participants received a smartphone along with the necessary instructions (verbally and written) to operate these devices. Subsequently, a trial run of the actual ESM questionnaires was conducted to affirm all questions were understood. Finally, participants completed computerized measures of current levels of depressive symptoms (PHQ-9). From the next day on, participants started the actual ESM protocol.

After one week of ESM, participants returned to the lab for a second group-session (T2). They returned the smartphones and completed the same computerized measurements as on T1. Additionally, we asked participants whether they had experienced any notable life-events during the past seven days, allowing us to take in account the possibility that these events had distorted their reported data, and thereby overshadowed potential markers of reactivity.

One month after the second lab session, participants received an e-mail requesting the completion of our computerized follow-up measures (PHQ-9), within 48 hours (T3). To minimize missing data and incomplete participations, it was stressed at the start of the study that participants would only be reimbursed after completing these last questionnaires.

### Statistical analysis

#### Differences in trajectories

As a first indicator of reactivity, we compared differences in the linear trajectories of the momentary emotional self-reports throughout the ESM week for every condition using mixed models. First, we averaged all ESM items in the positive, negative, and control condition per beep, creating a momentary measure of PA, NA, and CA per measurement occasion. Subsequently, these variables were modeled as function of time to examine possible temporal increases or decreases.

We used model Eq (1) to test whether significant changes in affect were present within conditions throughout the ESM procedure.

Level 1 $$AFFECT_{ij}=\beta_{0j}+\beta_{1j}\left(TIME_{ij}\right)+r_{ij}$$
Level 2 $$\begin{array}{l} \beta_{0j}=\gamma_{01}*\left(POSITIVE_{j}\right)+\gamma_{02}*\left(NEGATIVE_{j}\right)+\gamma_{03}*\left(CONTROL_{j}\right)+\upsilon_{0j}\\ \beta_{1j}=\gamma_{11}*\left(POSITIVE_{j}\right)+\gamma_{12}*\left(NEGATIVE_{j}\right)+\gamma_{13}*\left(CONTROL_{j}\right)+\upsilon_{1j} \end{array}$$ (1)

At the first level, the outcome AFFECT_*ij*_ represents mean levels of momentary affect at beep *i* for condition *j* (i.e., positive, negative or control), modelled as function of a random intercept (*β*_0j_), which represents the mean levels of momentary affect per condition, and a random slope (*β*_1j_), which reflects the effect of time throughout the ESM period (TIME_*ij*_). The residual was represented by *r*_ij_. At the second level, the positive, negative and control conditions were coded by three dummy variables (coded as 1 when the respective momentary survey at level 1 was associated with this condition, and 0 otherwise), omitting the intercept. Residuals were represented by *υ*_*ij*_.

Next, we assessed whether there were significant differences present between the slopes of the different conditions using the model Eq (2).

Level 1 $$AFFECT_{ij}=\beta_{0j}+\beta_{1j}\left(TIME_{ij}\right)+r_{ij}$$
Level 2 $$\begin{array}{l} \beta_{0j}=\gamma_{00}+\gamma_{01}*\left(CONDITION_{j}\right)+\upsilon_{0j}\\ \beta_{1j}=\gamma_{10}+\gamma_{11}*\left(CONDITION_{j}\right)+\upsilon_{1j} \end{array}$$ (2)

Differences between condition were examined by creating three data-subsets, each comprising two conditions, allowing us to exhaustively contrast all three conditions with one another. The same level 1 equation was used. At the second level, CONDITION_*j*_, represents one of the three conditions, coded categorically as a dummy variable, allowing us to assess whether including this variable in the equation yielded a significantly different slope in contrast to the intercept (*γ*_*10*_) alone, representing the opposing condition.

#### Changes in depressive symptom levels

A second indicator of reactivity was assessed by examining changes in levels of depressive symptoms throughout the study as a function of condition. This was achieved by comparing T1, T2 and T3 assessments of levels of depressive symptoms per condition using a mixed-model ANOVA.

First, we conducted a one-way between-subjects ANOVA to compare initial levels of depressive symptoms for the positive, negative and control condition. No significant differences were observed at T1, *F*(2, 87) = 2.71, *p* = .072. This indicates that, at the start of the study, participants in all three conditions experienced similar levels of depressive symptoms.

Second, we measured and compared the impact of the ESM procedure on participants’ PHQ-9 scores across all three conditions by conducting a mixed-model ANOVA. More specifically, for all conditions, we tested whether partaking in the ESM procedure entailed significant differences concerning depressive symptom levels throughout T1, T2 and T3.

These analyses were computed using SPSS (version 25.0; IBM Corp., 2017) and HLM (version 7.03; SSI Inc., 2013).

## Results

### Preliminary analyses

Overall compliance to the ESM questionnaires was high (*M* = 92.1%; *SD* = 6.8%; range: 66% - 100%) and did not differ significantly per condition, *F*(6, 83) = 0.44, *p* = .85. High compliance rates, combined with the fact that all participants provided data at all three timepoints, led us to include the total sample for analysis (*N* = 90). Distinguishing between participants who took part in the study in exchange for course credits and participants who took part on a voluntary basis did not yield contradictory conclusions, nor differences in compliance. Some participants had indicated they experienced notable life-events during the course of the study, yet removing these cases did again not alter our conclusions.

### Differences in trajectories

**Fig 1:**
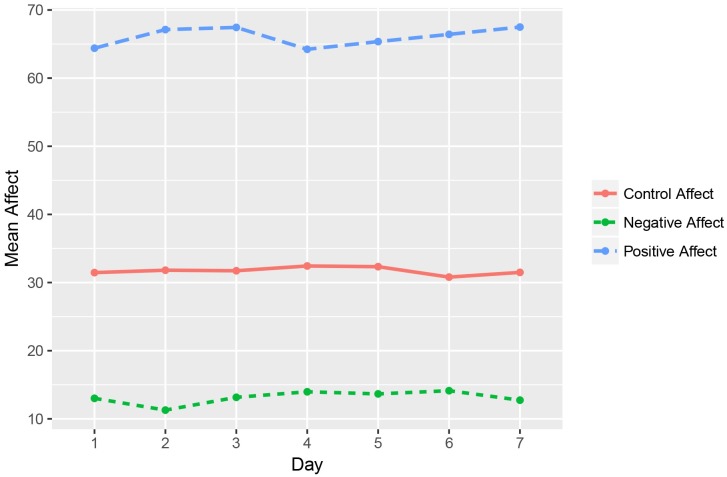
Trajectories of mean levels of momentary affect per condition during ESM.

The average trajectories for positive affect in the positive condition (PA), negative affect in the negative condition (NA), and ‘control’ affect in the control condition (CA) are visualized in Fig 1.

Results of the multilevel regression analyses are shown in Table 2. The various slopes indicated that there were no significant changes in the experience of affect within either the positive, negative or control condition, throughout the ESM procedure.

**Table 2:** Mixed models assessing the effect of time in each condition, and differences therein between conditions.

Variable	Intercept (*SE*)	Time slope (*SE*)	*p*-value time slope
Effect of time within conditions			
Positive condition	65.21 (1.803)	0.007 (0.01)	.568
Negative condition	12.84 (1.628)	0.005 (0.01)	.663
Control condition	31.98 (0.841)	0.003 (0.01)	.619
Differences between conditions			
Positive versus Negative Condition		0.002 (0.02)	.898
Positive versus Control Condition		0.004 (0.01)	.461
Negative versus Control Condition		0.002 (0.01)	.530

We also observed no significant differences between the slopes of the emotional trajectories of the positive and the negative condition, the positive and the control condition, or the negative and the control condition (Table 2).

### Changes in depressive symptom levels

**Fig 2:**
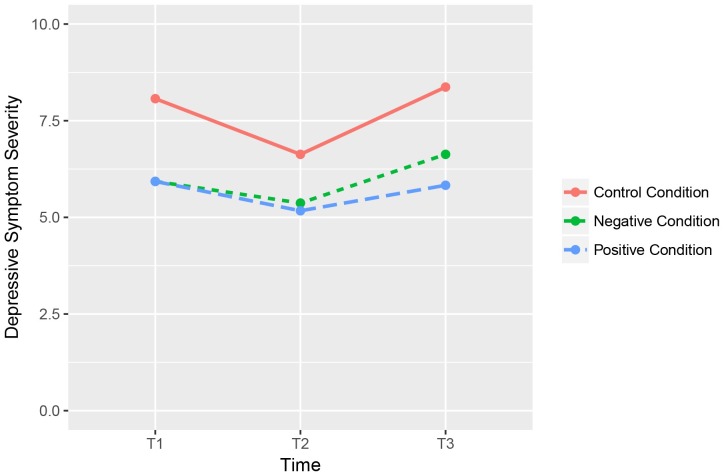
Differences in participants’ depressive symptom levels (as measured with the PHQ-9), before (T1), immediately after (T2), and one month after (T3) the ESM protocol, for each condition.

A visual representation of the changes in levels of depressive symptoms is shown in Fig 2.

We found a significant main effect of time, *F*(2, 174) = 8.112, *p* < .001). This indicates that depressive symptom scores differed significantly throughout the three different timepoints across all three conditions. Post-hoc contrast tests revealed that PHQ-9 scores significantly decreased between T1 and T2, *F*(1, 87) = 9.121, *p* = .003, and significantly increased back to baseline levels between T2 and T3, *F*(1, 87) = 18.151, *p* < .001. However, a critical Time x Condition interaction was not observed, *F*(4, 174) = .724, *p* = .577). This indicates that the general pattern regarding depressive symptom levels (i.e. a temporary decrease) did not differ significantly between conditions.

## Discussion

The aim of the present study was to assess potential reactivity effects of the repeated reporting on emotions in experience sampling research. As a typical ESM procedure involves the repeated awareness, labeling and communication of one’s feelings, we hypothesized that this protocol could (1) cause alterations in participants’ self-reported momentary emotions over time, and (2) indirectly influence people’s depressive symptom levels. To test this idea, we enrolled participants in a 7-day ESM protocol, reporting on their momentary experience of either positive, negative, or ‘control’ affective states. For each condition, we (1) investigated the corresponding emotional trajectory during ESM, and (2) differences in depressive symptom levels, before, immediately after and one month after the ESM procedure.

Regarding alterations in emotional trajectories, results did not confirm our expectations. Generally, no evidence was found that levels of self-reported affect systematically increased or decreased throughout the week of intensively reporting on emotional states. This was found for participants reporting positive as well as negative emotions as well as neutral internal states. These findings deviate from earlier research conducted by Conner and Reid [17] upon the subject of reactivity, who found changes in levels of momentary happiness during an ESM protocol. As these changes were moderated by one’s levels of trait negative affect, it could be the case that here, analogously, diverging emotional trajectories were moderated by baseline levels of depressive symptoms [17]. Upon further statistical analyses however, such moderation was not replicated, as both people high and low in baseline levels of depressive symptoms exhibited a similar emotional trajectory throughout the ESM protocol.

Also in terms of depressive symptom levels, reporting positive or negative emotions had little differential impact. We observed depressive symptom levels to decrease in all three conditions immediately after compared to before one week of ESM, but then to return to initial levels when measured at one-month follow-up. Such an immediate decrease in depressive symptom levels after reporting positive or negative emotions is in line with previous research [18–19]. Not in line with our expectations is that we also observed a decrease in depressive symptom levels in the control condition. A potential explanation of these findings might lie in the selection of the items that were being used in the control condition. These items–which were mostly bodily sensations–might have had emotional counterparts; for instance, feelings of hunger which went accompanied by feelings of frustration or irritation (e.g. feeling hangry) [23–24]. Therefore, we might speculate that participants in this condition, to some extent, still reported on emotional experiences. This could possibly explain why they experienced a temporary decrease in levels of depressive symptoms as well, conforming to the participants in the experimental conditions; in that, all three conditions might have experienced the beneficial effects that becoming aware, verbalizing and reporting on emotions entail. It was however very difficult to formulate internal subjective states that are completely independent from any emotional meaning or connotation. One might even argue that this is an impossible task due to the deeply-ingrained and omnipresent nature that characterizes our emotions. Another potential explanation is that this similar effect on depressive symptom levels in all three conditions reflect a type of Hawthorne effect [25]: it could be the case that the observed temporary decrease in levels of depressive symptoms was merely the result of participants taking part in the ESM experiment itself. In any case, the slight decrease in depressive symptoms did not last, as participants returned to pre-test levels at one-month follow-up in all three conditions. Overall, these results suggest no, or a very modest, impact on momentary emotional self-reports over time and levels of depressive symptoms caused by taking part in a 7-day ESM protocol.

### Limitations

Several limitations are present in this study. First, the sample of this study mostly comprised undergraduate students, limiting the generalizability of our results to broader samples.

Second, this study only looked at changes in levels of depressive symptoms and self-reported momentary affect as indicators of reactivity, limiting the inferences that could be made concerning reactivity to ESM in itself. Possible reactivity could still have been expressed in alternate manners and therefore remained undetected in this study. For example, we might consider the case that emotions might have been experienced differently than they would be had they not been one-sidedly reported by valence; that is, positive/negative emotions only. A within-subjects study-design in which emotions are firstly reported in a valence-based fashion followed by a mixed fashion, would therefore be an important avenue for future research.

Third, we acknowledge the rather limited time-frame in which the study was conducted; that is, one week of ESM. It is possible that reactivity to the ESM required a longer time-frame to manifest itself and therefore remained undetected. After all, the abovementioned studies in which reactivity was found, took place over significant larger periods of time (e.g. a 14-day protocol by Conner & Reid [17], a 30-day protocol by Broderick & Vikingstad [18], a 6-week protocol by Kramer et al. [19]). However, at the same time, we want to point out the rather intensive ESM protocol that characterized our study; in that, participants completed ten emotion-focused questionnaires, each containing ten items, for seven days, which sums up to (a maximum of) 700 emotion reports provided by each participant, which is far above the average in the abovementioned studies.

Lastly, while comparing effects of reporting on positive, negative, and non-emotions certainly held some explanatory value concerning reactivity, one could call it rather unconventional to make such between-condition inferences; in that, in these different conditions, different constructs were measured [26]. Therefore, further study-designs should be explored in which conclusions about changes in momentary affect could be made in more orthodox ways. For example, the within-subjects study-design mentioned above might be more appropriate here as well.

With this being the first study specifically designed to research reactivity to the ESM, we recommend to further explore these preliminary findings in future research while considering abovementioned limitations: Future studies building on these results should ideally include a more representative sample; a wider array of reactivity-measures; and a between-subjects study-design in which conclusion about changes in momentary affect could be made in a more conventional way. By applying these recommendations, more robust conclusions concerning the phenomenon of reactivity to the ESM could be made.

## Conclusion

To our knowledge, the current study was the first to explicitly address the question of reactivity to ESM methods in an experimental way. We examined possible indicators of reactivity by investigating (1) changes in the trajectories of the condition-specific, self-reported, emotional experiences throughout the ESM week as well as (2) changes in participants depressive symptom levels, throughout the study.

Results revealed (1) no significant changes in the trajectories of self-reported emotional experiences throughout the ESM week. Furthermore, although we observed (2) a significant decrease in depressive symptom levels immediately after the ESM protocol, these levels returned back to baseline one month later. Since this pattern was observed in both experimental conditions as well as in the control condition, ESM’s emotion-focused protocol did probably not lie at the basis of it.

## References

[1] Clark LA, Watson D. Tripartite model of anxiety and depression: Psychometric evidence and taxonomic implications. Journal of Abnormal Psychology. 1991 8;100(3):316–336. 10.1037/0021-843X.100.3.316 1918611

[2] Diener E, Suh EM, Lucas RE, Smith HL. Subjective Well-Being: Three Decades of Progress. 1999 3;125(2):276–302. 10.1037/0033-2909.125.2.276

[3] Zeidner M, Endler NS. Handbook of coping: Theory, research, applications. Oxford: John Wiley & Sons; 1996 752 p.

[4] Gross JJ, Thompson RA. Handbook of emotion regulation. 2nd ed New York: Guilford Press; 2007 668 p.

[5] Morris ME, Kathawala Q, Leen TK, Gorenstein EE, Guilak F, Labhard M, et al Mobile Therapy: Case Study Evaluations of a Cell Phone Application for Emotional Self-Awareness. J Med Internet Res. 2010 4;12(2):e10 10.2196/jmir.1371 20439251 PMC2885784

[6] Swinkels A, Guiliano TA. The measurement and conceptualization of mood awareness: monitoring and labeling one’ mood states. Personality and Social Psychology Bulletin. 1995 9; 21(9):934–949. 10.1177/0146167295219008

[7] Lieberman MD, Eisenberger NI, Crockett MJ, Tom SM, Pfeifer JH, Way BM. Putting feelings into words. Psychological Science. 2007 5;18(5):421–428. 10.1111/j.1467-9280.2007.01916.x 17576282

[8] Constantinou E, Van Den Houte M, Bogaerts K, Van Diest I, Van den Bergh O. Can words heal? Using affect Labeling to Reduce the Effects of Unpleasant Cues on Symptom Reporting in IBS Patients. Frontiers in psychology. 2014 7;5:807 10.3389/fpsyg.2014.00807 25101048 PMC4106456

[9] Kircanski K, Lieberman MD, Craske MG. Feelings Into Words: Contributions of Language to Exposure Therapy. Psychological Science. 2012 10;23:1086–1091. 10.1177/0956797612443830 22902568 PMC4721564

[10] Lieberman MD, Inagaki TK, Tabibnia G, Crockett MJ. Subjective Responses to Emotional Stimuli During Labeling, Reappraisal, and Distraction. Emotion. 2011 6;11(3):468–480. 10.1037/a0023503 21534661 PMC3444304

[11] Brans K, Van Mechelen I, Rimé B, Verduyn P. To share, or not to share? Examining the emotional consequences of social sharing in the case of anger and sadness. Emotion. 2014 12;14(6):1062–1071. 10.1037/a0037604 25151517

[12] Frattaroli J. Experimental disclosure and its moderators: A meta-analysis. Psychological Bulletin. 2006 11;132(6):823–865. 10.1037/0033-2909.132.6.823 17073523

[13] Thomassin K, Morelen D, Suveg C. Emotion reporting using electronic diaries reduces anxiety symptoms in girls with emotion dysregulation. Journal of Contemporary Psychotherapy. 2012 12;42(4):207–213. 10.1007/s10879-012-9205-9

[14] Radcliffe AM, Lumley MA, Kendall J, Stevenson JK, Beltran J. Written Emotional Disclosure: Testing Whether Social Disclosure Matters. Journal of Social and Clinical Psychology. 2007 3;26(3):362–384. 10.1521/jscp.2007.26.3.362 20824150 PMC2932452

[15] Kahneman D, Krueger AB, Schkade DA, Schwars N, Stone AA. A survey method for characterizing daily life experience: The day reconstruction method. Science. 2004 10;306(5702):1776–1780. 10.1126/science.1103572 15576620

[16] Ebner-Priemer UW, Trull TJ. Ecological Momentary Assessment of Mood Disorders and Mood Dysregulation. Psychological Bulletin. 2009 12;125(2):276–302. 10.1037/a0017075 19947781

[17] Conner TS, Reid KA. Effects of intensive mobile happiness reporting in daily life. Social Psychological and Personality Science. 2011 8;3(3):315–323. 10.1177/1948550611419677

[18] Broderick JE, Vikingstad G. Frequent assessment of negative symptoms does not induce depressed mood. Journal of Clinical Psychology in Medical Settings. 2008 8;15(4):296–300. 10.1007/s10880-008-9127-6 19104986 PMC2730150

[19] Kramer I, Simons CJP, Hartmann JA, Menne-Lothmann C, Viechtbauer W, Peeters F, et al A therapeutic application of the experience sampling method in the treatment of depression: a randomized controlled trial. World Psychiatry. 2014 2;13(1):68–77. 10.1002/wps.20090 24497255 PMC3918026

[20] Vachon H, Bourbousson M, Deschamps T, Doron J, Bulteau S, Sauvaget A, et al Repeated self-evaluations may involve familiarization: An exploratory study related to Ecological Momentary Assessment designs in patients with major depressive disorder. Psychiatry Research. 2016 11;245:99–104. 10.1016/j.psychres.2016.08.034 27541343

[21] Nezlek JB. A practical guide to understanding reliability in studies of within-person variability. Journal of Research in Personality. 2017 8;69:149–155. 10.1016/j.jrp.2016.06.020

[22] Kroenke K, Spitzer RL, Williams JBW. The PHQ-9 Validity of a Brief Depression Severity Measure. Journal of General Internal Medicine. 2001 9;16(9):606–613. 10.1046/j.1525-1497.2001.016009606.x 11556941 PMC1495268

[23] Bushman BJ, DeWall CN, Pond RS, Hanus MD. Low glucose relates to greater aggression in married couples. PNAS. 2014 4;111(17):6254–6257. 10.1073/pnas.1400619111 24733932 PMC4035998

[24] Danziger S, Levav J, Avnaim-Pesso L. Extraneous factors in judicial decisions. PNAS. 2011 4;108(17):6889–6892. 10.1073/pnas.1018033108 21482790 PMC3084045

[25] Landsberger HA. Hawthorne revisited. Ithaca: Cornell University; 1958 119 p.

[26] Diener E, Larsen RJ, Levine S, Emmons RA. Intensity and Frequency: Dimensions Underlying Positive and Negative Affect. 2004 5;48(5):1253–65. 10.1037/0022-3514.48.5.1253 3998989

